# Automatic Detection of K-Complexes Using the Cohen Class Recursiveness and Reallocation Method and Deep Neural Networks with EEG Signals

**DOI:** 10.3390/s21217230

**Published:** 2021-10-30

**Authors:** Catalin Dumitrescu, Ilona-Madalina Costea, Angel-Ciprian Cormos, Augustin Semenescu

**Affiliations:** 1 Department Telematics and Electronics for Transports, University “Politehnica” of Bucharest, 060042 Bucharest, Romania; ilona.costea@upb.ro (I.-M.C.); angel.cormos@upb.ro (A.-C.C.); 2 Department Engineering and Management for Transports, University “Politehnica” of Bucharest, 060042 Bucharest, Romania; augustin.semenescu@upb.ro

**Keywords:** K-complexes, sleep disorders, Cohen class, sleep stage, classification, deep neural networks

## Abstract

Evoked and spontaneous K-complexes are thought to be involved in sleep protection, but their role as biomarkers is still under debate. K-complexes have two major functions: first, they suppress cortical arousal in response to stimuli that the sleeping brain evaluates to avoid signaling danger; and second, they help strengthen memory. K-complexes also play an important role in the analysis of sleep quality, in the detection of diseases associated with sleep disorders, and as biomarkers for the detection of Alzheimer’s and Parkinson’s diseases. Detecting K-complexes is relatively difficult, as reliable methods of identifying this complex cannot be found in the literature. In this paper, we propose a new method for the automatic detection of K-complexes combining the method of recursion and reallocation of the Cohen class and the deep neural networks, obtaining a recursive strategy aimed at increasing the percentage of classification and reducing the computation time required to detect K-complexes by applying the proposed methods.

## 1. Introduction

It is hard to explain the mechanism that triggers sleep. Science can only put forth some hypotheses, but there is still no certainty. Sleep can only be studied in its external manifestations, while the causes that generate it remain unknown. The mechanisms that maintain the state of wakefulness and the transition from wakefulness to sleep sparked the interest of scholars many centuries ago.

However, since the first theories were laid down by Alcmaeon of Croton and Aristotle, there has not been much progress in this field. It is only in the past 80 years that a review of old ideas about wakefulness and sleep has begun, largely due to the enhancement of research tools that have allowed for the precise exploration of deep brain structure and the recording of electrical brain activity down to the level of a single cell. Existing theories on sleep and wakefulness feature great diversity but can be grouped into two categories: humors-related, including particularly old and very old theories, and *neural* theories, specific to the modern age and based on experimental investigations.

The study of electroencephalograms (EEG) began in the 1930s as the first attempt at classifying human cerebral activities during sleep. Only after a long period of analytical interpretation were efforts eventually directed towards the analysis of the transient phenomena occurring during sleep. At present, the main objective lies in understanding the mechanisms that generate sleep and the psychological role of electrical activity in the brain.

The applicability of the method proposed in this article focuses on the analysis, detection, and classification of the K-complex, which is a transient brain wave in the microstructure of the electroencephalogram (EEG) of sleep. Detection of the K-complex brain wave is difficult because during sleep other nonstationary phenomena occur, including an increase in delta waves, which looks very similar to the K-complex. The K-complex investigation method proposed in this article allows for an automated system to detect and classify—with very good probability—the aperiodic K-complex that occurs in the second stage of EEG sleep. According to the literature, the K-complex has an important role in the study of sleep [1,2,3,4], the detection of diseases associated with sleep disorders [5,6,7,8,9,10,11,12,13,14,15,16,17], and recently has been suggested as a biomarker for predicting the occurrence of Parkinson’s [18,19,20,21,22,23,24,25,26,27] and Alzheimer’s diseases [28,29,30,31,32,33,34,35,36,37].

The results reported in a series of papers on the study of sleep electroencephalograms justify the interest in using time–frequency-pooled analyses that can characterize the transient phenomena that occur during the stages of sleep [38,39,40,41,42].

The two-dimensional representations in the time–frequency domain provide a powerful signal analysis tool that has the advantage of making it possible to highlight certain “hidden” properties of the signals. As far as the system of analysis is concerned, the main interest lies in analyzing signals at the lowest level, comparable to the noise made by the EEG apparatus. This is why time–frequency analyses should be performed on signals affected by noise, the signal/noise ratio being of particular importance for evaluating the parameters of the analyzed signal. It is this method of representation and analysis of transient signals that we have resorted to in order to develop a K-complex detector. The proposed methodology for the synthesis of imposed structure detectors has wider applicability, for example, in the processing of vocal signals.

Form recognition has become a growing field in both theory and application, being, on the one hand, an advanced form of information processing, and on the other, a component of artificial intelligence. Many mathematical methods have been proposed to solve the problem of form recognition, and have been grouped into two categories—statistical–decisional and syntactic–structural ones [43,44,45,46,47,48,49,50].

The theory of detection aims at making an optimal decision, chosen from a finite number of possible alternatives, with the purpose of obtaining a random sample. In particular, when detecting a disturbed noise signal, the decisions taken into consideration envisage the validation of one of the following hypotheses: “observation *x(t)* consists of noise” or “signal *s(t)* is present in observation *x(t)*”. The criterion that sets the objectives of the detection structure in optimal terms may then, for example, be to minimize the average cost of a decision or even increase the probability of detecting signal *s(t)* under the condition of an imposed false alarm probability. For these two criteria, one has to demonstrate that the optimal value is achieved by choosing the plausibility ratio for the *Λ* detection statistics. Comparing it to a *λ*_0_ threshold then generates the decision: signal *s(t)* is assumed to be present in observation *x(t)* if *Λ* is superior to *λ*_0_ and absent otherwise. One refers to it in this case as frame detection, especially since no constraint acts upon the structure of detection statistics, the latter relying only on the choice of a rule and the knowledge/implementation of plausibility laws [51]. At the same time, in a large number of situations, the information provided by the statistical properties of the sample for each hypothesis is not available, which makes the previous study impossible. Under these circumstances, a possible but suboptimal case consists of defining in advance the nature of the detection statistics, then optimizing the characteristic parameters according to a given criterion. For this reason, this study is known as imposed structure detection [52].

Under these circumstances, the problems encountered during the development of a detection rule are generally related to the difficulty of answering the following questions:

- How to choose the pattern/model of detection?
- What are the optimal criteria for determining the characteristic parameters of this model? What optimization procedure is being adopted?

In this article, we propose a new approach to the analysis, recognition, and classification of K-complexes based on the application of recursion properties to Cohen class distributions modified by the reallocation method, developing a recursive strategy to capitalize the reallocation algorithm for time–frequency representations. We also demonstrate the advantage of the size of the time support of the parameterization function for Cohen class distributions, but especially for the Wigner–Ville distribution, proposing an algorithm for using recursion in the reallocation process of the Wigner–Ville distribution spectrogram. Next, we established the necessary and sufficient conditions allowed by the analysis windows and the parameterization function, so that the Cohen class distributions could be used after the introduction of recursion and we extended these conditions for the distributions modified by the reallocation method. We used the results obtained from the Wigner–Ville distribution to train a convolutional neural network (deep learning), which we trained with the Calibrated Stochastic Gradient Descend (CSGD) optimization algorithm because it updates the parameters for each training stage. SGD also eliminates the redundant calculations that occur for large datasets and EEG records, by performing frequent updates. Another advantage of SGD is the speed of calculations. The test results showed a detection percentage of 98.30%.

The rest of the paper is structured as follows: it presents the model of analysis and detection of K-complexes in Section 2.1, the EEG microstructure of sleep in Section 2.2, a comparative analysis of Cohen class energy distributions in Section 2.3, the new algorithm for recursiveness and reallocation in the Section 2.4 and Section 2.5, and the results obtained by the new method proposed together with the deep neural network used in Section 3.

## 2. Materials and Methods

### 2.1. Proposed Methods

**Figure 1:**
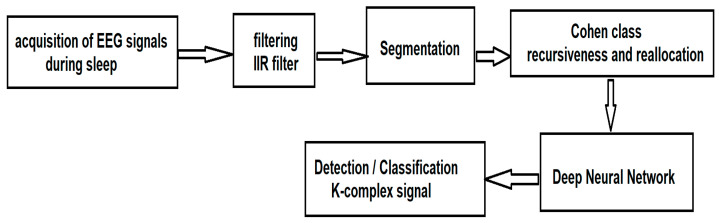
The architecture of the proposed method for the detection of K-complex brain waves.

Once the signals have been acquired, according to the traditional method of acquiring EEG signals during sleep, they are processed according to the sequence of steps illustrated in Figure 1.

First, the EEG signals must be filtered. This step is imperative because the acquired data overlap a parasitic signal of 50 Hz coming from the power supply network that can affect the useful signal, making the classification almost impossible. To eliminate the area of network noise, an Infinite Impulse Response (IIR) notch filter, implemented in MATLAB, was used. The alternative to the IIR filter is the Finite Impulse Response (FIR) filter. The differences include:

- IIR has a faster response time than FIR [53];
- IIR phases out different components frequently; it is different from FIR, which generates a constant phase shift (IIR has a nonlinear phase and FIR has a linear phase) [53];
- FIR is always stable, while IIR is sometimes unstable [53].

Since the information of interest (through which the class will be made) is not in the phase component but in the frequency component, we chose for this first stage of processing the IIR filter. After segmentation, the Wigner–Ville algorithm from the Cohen class is applied, for which we introduce a new method of recursiveness and reallocation of the spectrum so as to extract the fundamental parameters that characterize the K-complexes. After extracting the characteristics, we use a deep neural network for the automatic detection and classification of K-complexes.

### 2.2. Microstructure of Sleep EEG

**Figure 2:**
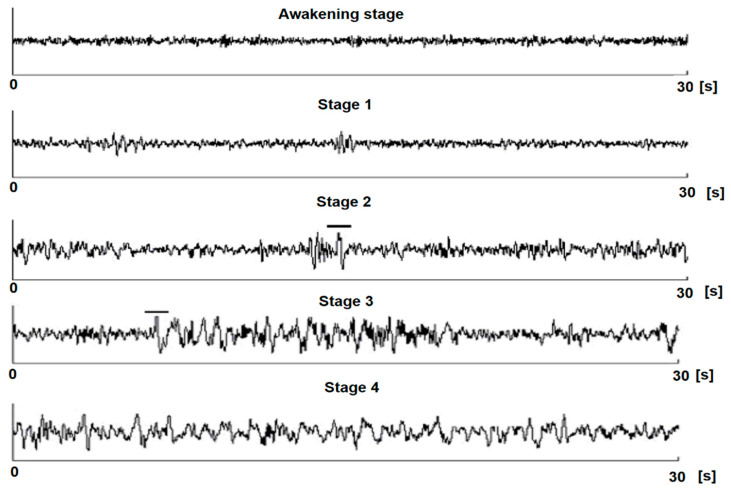
Sleep stage [49].

According to this classification, the four stages of sleep, represented in Figure 2, are as follows [49]:

- Stage 1: In this stage, we have smooth sleep: we easily pass from the waking state to the sleep state and can be awakened by even the slightest noise. Our eyes move very slowly and muscle activity slows down. We also experience sudden, involuntary muscle contractions called myoclonus, often preceded by a feeling of falling into emptiness. People awakened from this stage often remember fragmented visual images.
- Stage 2: When we enter the second stage of sleep, eye movements stop, the heart begins to beat slower, the muscles relax, and the body temperature drops. Also, brain waves (fluctuations in electrical activity, which can be measured by electrodes) become slower and a series of occasional fast waves called sleep spindles appear. Basically, the body prepares for deep sleep.
- Stage 3: Stage 3 of sleep is characterized by extremely slow brain waves and the lack of any eye movement or muscle spasms, which promotes deep sleep. Once we reach this stage, it is very difficult to wake up. People awakened during deep sleep do not immediately adapt to reality and, for a few minutes, are dizzy and disoriented.
- Stage 4: In this stage, the heart rate, breathing, and eye movements become faster and faster. The brain becomes more active, processing the things we learned during the day to help us form memories. Usually, during REM (Rapid Eye Movement) sleep, people dream; that is why those who are awakened at this stage often tell bizarre and illogical stories of what they experienced.

Sleep is of two types, each with its own characteristics:

(1) REM (rapid eye movement);
(2) non-REM, with four depth stages, 1, 2, 3, and 4.

When we fall asleep, we enter a state of non-REM sleep, in different stages; then we go into a state of REM sleep (rapid eye movement) characterized by rapid and simultaneous movements of both eyes, hence the name. Research studies show that the two types of sleep alternate with each other and take place in the form of cycles. The duration of these cycles is about 70–100 min (the first non-REM–REM cycle), the next cycles being longer, 90–120 min. Also, the passage through all stages of non-REM sleep is not observed. In the last period of sleep, morning sleep, the duration of REM sleep increases. It is not known exactly why these phases of sleep alternate. What is known is that there is a higher proportion of non-REM sleep (about 80%) compared to REM sleep (about 20–25%) [54].

The K-complex, together with the sleep cycles, is one of the main “markers” of sleep onset as it appears in stage 2.

The K-complex can have different morphologies, which can appear every fourth second, as follows: isolated K-complex, without spindle; K-complex and spindle insulated; K-complex pair; and K-complex with negatively flattened component. A K-complex is an EEG waveform that occurs in stage 2 of REM sleep. It is “the biggest event in a healthy person,” being more common in the first sleep cycles. The K-complexes have two proposed functions: first, to suppress cortical arousal in response to stimuli that the sleeping brain does not evaluate to signal a danger, and second, to support sleep-based memory consolidation. The K-complex consists of a short peak of high negative voltage, usually greater than 100 μV, followed by a slower positive complex around 350–550 ms and 900 ms at the final negative peak. The K-complexes occur approximately every 1.0 to 1.7 min and are often followed by bursts of sleep spindles. These occur spontaneously, but also in response to external stimuli, such as sounds, touching the skin, and internal stimuli, such as inspiratory interruptions. They are generated in diffuse cortical locations, although they tend to predominate over the frontal parts of the brain. Both the K-complex and the activity of delta waves in stage 2 sleep create a slow wave of 0.8 Hz and delta oscillations (1.6–4.0 Hz). However, their topographic distribution is different and the delta power of the K-complexes is higher. They are created by the diffuse appearance in the cortical areas of the external dendritic currents in the middle (III) and (I) starts of the cerebral cortex. This is accompanied by a decrease in the broadband power of the EEG, including the activity of gamma waves. This produces “down-states” of neural silence in which the activity of the neural network is reduced.

**Figure 3:**
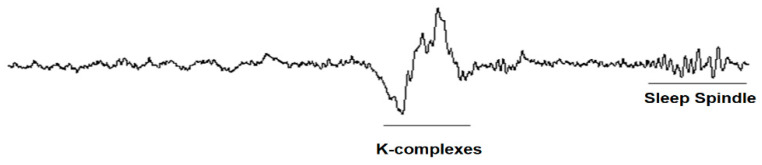
Brain waves: K-complex and sleep spindle [49].

**Figure 4:**
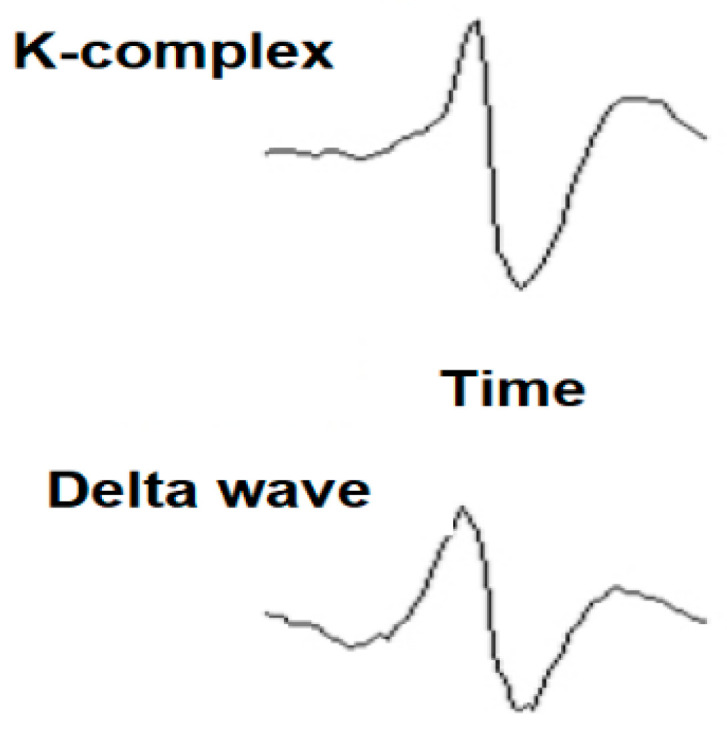
Waveforms characteristic of the K-complex and delta waves that occur during sleep [49].

The K-complex, sleep spindle and delta wave are shown in Figure 3 and Figure 4.

### 2.3. Comparative Analysis of Cohen Class Energy Distributions

As this is a comparative study that resorts to time–frequency representations, a linear frequency modulated pulse was used, of unit amplitude, and the frequency, rated/normed at sampling frequency, was increased linearly from 0.1 to 0.3. The signal length corresponds to 512 samples [55]. Table 1 presents the Cohen-class frequency–time representations and associated core functions.

**Figure 5:**
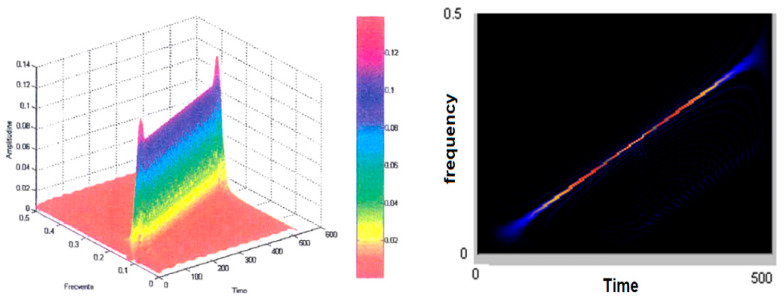
The pseudo-Wigner–Ville representation of the test signal.

Figure 5 (left) shows the smoothed-out Wigner–Ville pseudodistribution applied for the test signal. The dimensions of the smoothing windows for the simultaneous analysis in the time and frequency axes are 64 points and the Fourier transform is calculated at 512 points. It is noted that this “knife blade” representation preserves the temporal and frequency support and provides a precise picture of how the signal energy is distributed along the time–frequency plane. Figure 5 (right) shows the time–frequency plane viewed from above at an angle of 90°. It may be seen how thin the “energy blade” of the test signal is and how sharply it comes into prominence against the remaining background. It has been found that this has reduced the representation’s resolution in both time and frequency. Another effect of dimming the windows and the FFT dimension has been an increase in the amplitude of the secondary lobes. The advantages of using small-scale analytical windows include better preservation of the time and frequency supports, as well as a reduction in the calculation time. Even under these conditions, one may determine on whose signal type basis such representation was made in case the latter is not known a priori. Good enough estimates may also be made of the parameters of the signal that originated the transformation. The analysis window was in both cases of the Hamming type.

**Figure 6:**
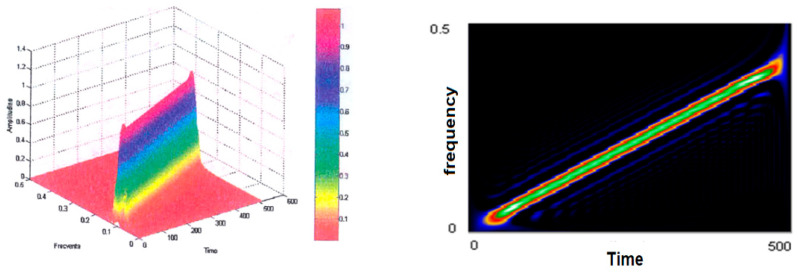
Born–Jordan representation of the test signal.

The test signal will be further analyzed using other Cohen class time–frequency representations, such as the Born–Jordan distribution represented in Figure 6, for which Hamming-type windows were used. Compared to the Wigner–Ville distribution, it is similar in that it looks like a somewhat thicker “knife blade,” with a lower resolution, but the secondary lobes are almost unnoticed when the window size *H* is equal to 64; when the window size decreases to 32, the resolution decreases and the secondary lobes become visible.

**Figure 7:**
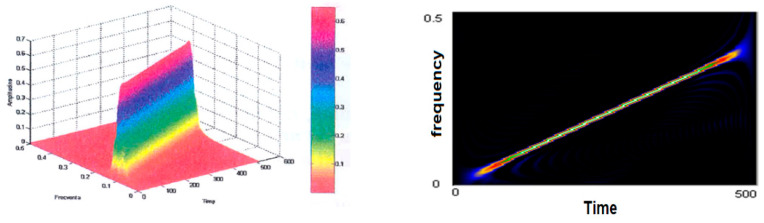
Choi–Williams representation of the test signal.

Figure 7 contains the Choi–Williams distributions of the linear variable frequency signal for a Hamming window sized 64. A first observation is related to the positive sign of the distribution and its large amplitude. The resolution of the representation is almost as good as the smooth Wigner–Ville pseudo distribution. Interestingly, the secondary lobes have not become visible for any of the dimensions of the analysis window. Instead, some disturbances perpendicular to the time axis and wide frequency support appeared at the signal edge, which are more pronounced when the size of the smoothing window is small.

**Figure 8:**
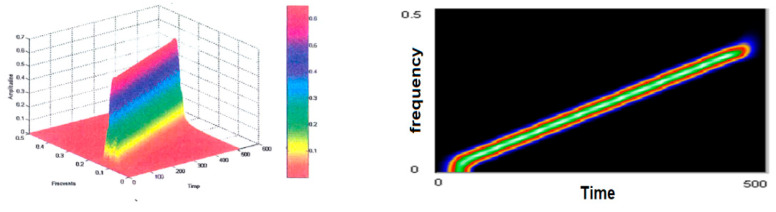
Zao–Mark–Atlas representation of test signal.

The Zao–Mark–Atlas distribution of the test signal is shown in Figure 8. As a first conclusion, the amplitude of the representation is lower that that of the other representations studied so far.

The resolution is very good, compared to the other distributions.

**Figure 9:**
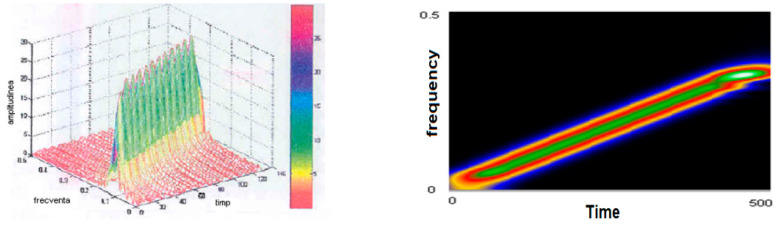
STFT spectrogram of the test signal.

The decrease in the size of the analytical window (to 32 vs. 64) led to the emergence of the secondary lobes, as well as to disturbances in the signal edges. We left to last the signal analysis made with the help of the spectrogram (Figure 9), which is also a Cohen class distribution; it had the poorest performance but was the simplest of all. The window used was rectangular, size 64.

From the analysis of a test signal based on time–frequency representations in the Cohen class, one may draw some conclusions:

- of all the time–frequency distributions investigated, the spectrogram provides the temporal resolution at the lowest frequency and the Zao–Mark–Atlas distribution features the smallest amplitude;
- the Choi–Williams distribution is positive and has the largest amplitude;
- the Born–Jordan distribution features almost no secondary lobes;
- the choice of analytical windows (Blackman, Hanning, Hamming, Bartlett, or rectangular) influences the temporal and frequency resolutions as well as secondary lobe levels.

In the wake of our investigations, some important aspects may be highlighted with respect to the use of signal analysis based on time–frequency distributions in the Cohen class, namely:

- the energy structure of the analyzed signals may be fairly accurately identified and located in the time–frequency plane;
- when the type, duration, frequency, and timing of signals are not known in advance, they may be estimated by using time–frequency distributions;
- one can thus foresee the possibility of implementing these analytical algorithms in the systems for identifying the EEG transient signals;
- even in the case of signals covering the same spectral range, generated by different sources, the time–frequency distributions allow each of them to be highlighted;
- one may set up databases that are useful for identifying EEG transient signals as their “signature” can be individualized by using time–frequency representations.

For the analysis of EEG signals in order to detect and classify K-complexes, we will use pseudo-Wigner–Ville reallocated distributions.

### 2.4. Recursive Implementation of Cohen Representations

Section 2.3 deals with practical problems that depend on the calculus of representations, a lesser subject if we consider that the development of rapid and elegant economic algorithms has widened and continues to broaden the applicability domains of the time–frequency analysis.

Two methods may be identified in this area: the first method envisages the limitation of the large volume of calculations. For this purpose, some authors look for an optimal ordering of operators [56,57,58]. Other authors, on the other hand, choose the symmetry properties of the instant autocorrelation function and some parameterization functions [59,60]. Finally, the distribution of the Cohen class may become the object of a division into a weighted sum of spectrograms, from which only the most significant terms have been retained.

Except for this latter situation, which, at most, can only provide an approximation of the representation, the above methods lead to a substantial reduction of the calculation time. Because of this, some of them are systematically associated with recursive implementation algorithms that are covered by the second method.

*Recursiveness and reallocation method*

The improvement of the contrast function for time–frequency representations is an important step for the analysis of nonstationary signals. That is why numerous solutions have been proposed, including the reallocation method. In this section, we propose a recursive strategy that aims at reducing the computation time required. Thus, we are interested in introducing recursiveness into the spectrogram reallocation process, then to the pseudo-Wigner–Wille distributions.

(a) Pseudo-Wigner–Ville reallocated distributions (Recursiveness and reallocation operators)

In [55], the authors formulated the relationships of reallocation operators associated with *C_x_* Cohen class distributions as follows:

(1) $$\hat{n}[n,v;x]=n\frac{C_{X}[n,v;T\varphi )}{C_{X}[n,v;\varphi )},$$

(2) $$\hat{v}[n,v;x)=v+j\frac{C_{X}[n,v;D\phi )}{C_{X}[n,v;\phi )},$$

where $T\phi_{TR}[m,l]=m\phi_{TR}[m,l]$ and $D\phi_{TR}[m,l]$ are the partial derivative of $\partial \phi_{TR}(t,\tau )/\partial \tau$ evaluated in (*m, l*). By adopting the same method as for the reallocated spectrogram, it may be shown that *C_x_*[.;*DΦ*) and *C_x_*[.;*TΦ*) may be evaluated recursively when *C_x_*[.;*Φ*) verifies the direct recursiveness relation (Equation (3)):

(3) $$C_{x}[n,v;\phi ]=\alpha c_{x}[n-l,v;\phi ]+c\alpha^{-M}C_{x}[n+m,v;\varphi ]-c\alpha^{M+1}C_{x}[n-M-l,v;\varphi ]$$

To reduce the complexity of the various recursive relationships presented, we have confined the present study only to the cases of pseudo-Wille distribution.

**Theorem** **1.** *Let Φ belong to the class of parameterization functions* $\xi ({\tilde{W}}_{X})$ *with separable variables that allow a direct recursive implementation of the pseudo-Wille–Ville distributions. The following relationships determine the direct and indirect recursiveness of W_x_[.;DΦ] and* ${\tilde{W}}_{x}$ *[.;TΦ]:*

(4) $${\tilde{W}}_{X}[n,v;D\phi )=\alpha {\tilde{W}}_{X}[n-1,v;D\phi )+\sum_{l=1-L}^{l-1}n[n,l)e^{-j4\pi vl}\;\mathrm{with}\;\eta [n,l]=D\phi_{TR}[M,l]R_{X}[n+M,l]-\alpha D\phi_{TR}[-M,l]R_{X}[n-M-1,l].$$

(5) $${\tilde{W}}_{X}[n,v;T\phi )=\alpha ({\tilde{W}}_{X}[n-1,v;T\phi )-{\tilde{W}}_{X}[n-1,v;\phi )+\sum_{l=1-L}^{l-1}\psi [m,l)e^{-j4\pi vl}\;\mathrm{with}\;\psi [n,l]=M\phi_{TR}[M,l]R_{X}[n+M,l]-\alpha (M+1)\phi_{TR}[-M,l]R_{X}[n-M-1,l]$$

*Equation (4) results if* *Φ* *belongs to* *ξ(* ${\tilde{W}}_{x}$ *); then DΦ also belongs to ξ(* ${\tilde{W}}_{x}$ *). Consequently, the direct recursive relation*

(6) $${\mathrm{PWL}}_{X}[n,v;\phi ]=\alpha {\mathrm{PWL}}_{X}[N-1,V;\phi ]+\sum_{L=-\infty }^{+\infty }k[n,l]e^{-j4\pi vl}$$

*remains valid for the distribution* ${\tilde{W}}_{x}$ *[.;*DΦ*).*

A simple calculation allows us to verify Equation (5). Note that the association of several recursive processes of the same type as Equations (4) and (5) also allows for the extension of the recursive strategy that we have previously presented to the class of parameterization functions *ξ_ext_*(${\tilde{W}}_{x}$). In this case, use is made of the linearity property of the Fourier transform.

(b) Performances

The kernels of the separable variables g[l] h [m], written as *gh*, which we use to demonstrate interest in the recursive strategy, verify the Hermitian symmetry property:

(7) g[l]h[m]∗=g[l]h[−m]

This results in:

(8) (Dgh[l,m))*=Dgh[l,−m] si (Tgh[l,m])*=Tgh[l,−m],

which allows us to rewrite the pseudo-Wigner–Ville distributions associated with nuclei *Tgh* şi *Dgh* as follows:

(9) W˜X[n,v;Tgh)=2Re{Ξx[n,v;Tgh)}−∑m=−MMTgh(0,m]Rx[n+m,0]

(10) W˜X[n,v;Dgh)=2Im{Ξx[n,v;Dgh)}−∑m=−MMDgh[0,m]Rx(n+m,0], 

where

(11) $$\Xi_{x}[n,v;\phi )=\sum_{l=0}^{l-1}\sum_{m=-M}^{M}\phi [l,m]R_{x}[n+m,l]e^{-j4\pi vl}.$$

**Figure 10:**
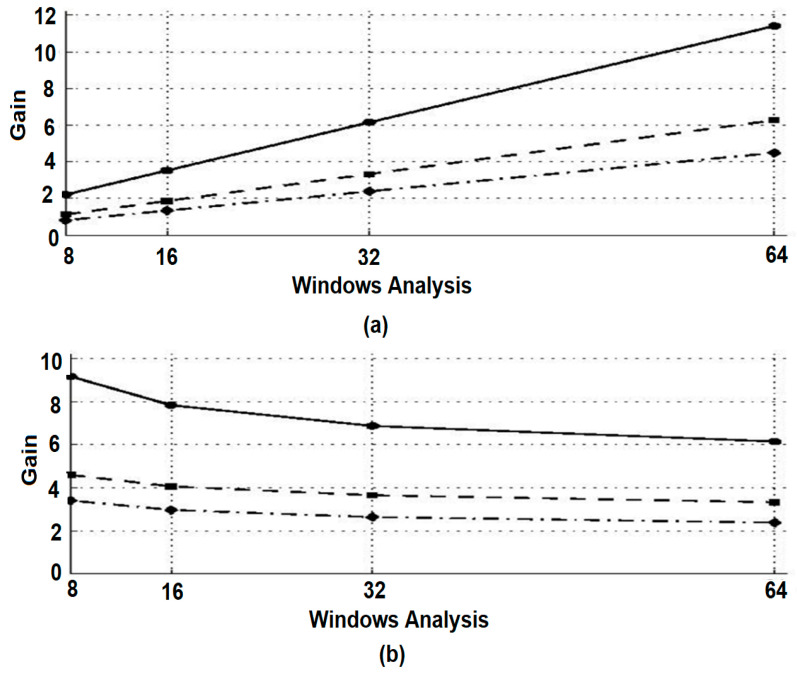
Estimation of time gain by using recursive calculation applied to the classical method for evaluating the re-allocated pseudo-Wigner–Ville distribution. Different time windows *h* are analyzed: rectangular (●), semi-sinusoidal (■), Hamming, and Hanning (♦). (**a**) Temporal window *h* of semilength *l* that is set at 64. (**b***)* Frequency window *g* with semilength M set at 32.

The relationships retain the general characteristics found in Equations (4) and (5). If the adopted strategy is classical or recursive, the use of Equations (9)–(11) allows for a reduction in the time needed to calculate distributions [., *Tgh*] and [., *Dgh*] and thus the reallocation operators. Finally, in order to compare the two methods under the most favorable conditions, the calculations of algorithmic complexity mentioned were made on the basis of these relationships. These results emphasize the fact that, for the recursive algorithm, complexity is independent of the temporal support [−M, M] of the temporal window h. This represents an advantage of the proposed method, as shown in Figure 10a. In contrast, Figure 10b shows that the calculation time gain decreases when the window width g increases for a given time window *h*.

**Figure 11:**
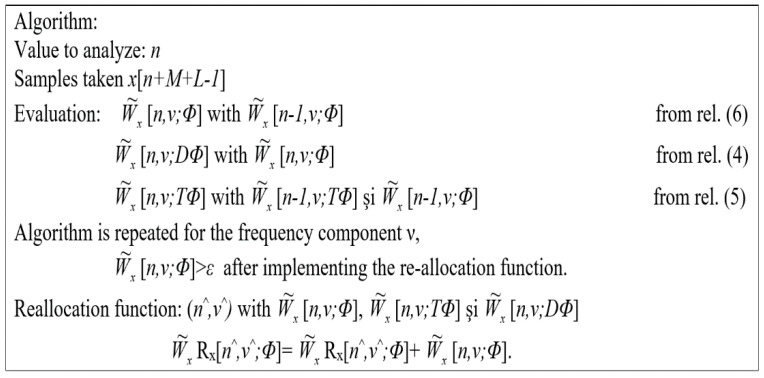
Algorithm with recursive strategy for the evaluation of the reallocated pseudo-Wigner–Ville distribution.

Finally, it is noted that the calculation time gain is not affected by the number of samples ${\tilde{W}}_{x}$[n,v;gh) reallocated at each moment *n*, and the calculation of the reallocation algorithm function shown in Figure 11 requires a maximum of 3N additions and 2N multiplications. This specification is valid for the reallocated spectrogram.

### 2.5. Deep Neuronal Network

To see the separability of the classes, various architectures of deep neural networks were used. As previously mentioned, the features provided as input data consist of the correlations extracted with the Cohen class algorithm. Thus, the networks were tested with a number of hidden layers ranging from 1 to 7. Also, the number of neurons on each layer varied between 8 and 256 (more precisely, 8, 16, 32, 128, 256 neurons per layer were used). The activations tested were sigmoid, tanh, and arctan. They have been used due to the fact that they are known to have good results, especially for class problems [61,62], but also estimation problems [63].

Results with activation functions that deny components’ negatives (e.g., ReLU, Leaky ReLU) were not used in this paper because they provided random responses (an accuracy of around 50%). One possible reason for this is the phenomenon called dying ReLU. It refers to the fact that if too many weights take values below 0 the activation result will be 0, so data discrimination becomes impossible.

The optimization technique used was Stochastic Gradient Descent (SGD). This algorithm is used in the field of deep neural networks and generally provides satisfactory results [64,65,66,67]. The proposed method is a simple one from GD (Gradient Descent) in the sense that, instead of calculating the gradient for the whole lot at each iteration, the gradient is calculated only for a randomly chosen value from the lot [68]. Therefore, the learning process is stochastic and depends on these chosen values. In this way, it is desired that Equation (12) (3.8) for GD behave relatively similarly to Equation (13) (3.9) for SGD, having the advantage of a much smaller calculation volume:

(12) $$w_{t+1}=w_{t}-\eta \frac{1}{n}\sum_{i=1}^{n}\nabla_{w}ℒ\left(z_{i},w_{t}\right)$$

(13) $$w_{t+1}=w_{t}-\eta \nabla_{w}ℒ\left(z_{t},w_{t}\right).\;$$

In these formulas, z refers to the pair (x; y), meaning the input value (associated output value), and $z_{t}$ is the pair chosen at time *t*.

## 3. Results

Due to the relatively small size of the database, we used a batch size of value 1 (only one example was evaluated for updating the parameters). In addition, to ensure that all architectures were tested under the same conditions, the number of epochs was always 100. The tests were performed in two stages.

In the first stage, the database was divided into two: the training part with a size of 350 and the test part with a size of 39, equivalent to a division of 90% and 10%. During training, the purpose of the network is to determine the weights that allocate the ideal labels to the input values. The data that the network learns are called the training batch. In order to test the possibility of generalizing the found function, some values are kept separately (called the test batch) and passed through the network. If the results are good, we can say that the network has learned and is able to classify the new data.

At this stage, different deep neural network architectures were tested. During the tests, the hyperparameters of the system were modified. More precisely, we experimented with various values for the number of neurons, the layer, and various activation functions—tanh, arctan, and sigmoid. The three activation functions were chosen because they have an S shape, which increases the separability of the input data.

The neural architecture tested for the tanh function had five layers and 16 neurons per layer, and the best cost recorded on the test was 0.32 with an accuracy of 90.18%.

The results obtained for the tanh activation function are presented in Table 2.

The next activation function tested was sigmoid, and the best cost on the test batch was 0.35 at an accuracy of 94.65%. It has also been observed that, with the increase in the number of hidden layers and even the number of neurons, the network begins to stop learning and gives random results for training data. The results obtained for the sigmoid activation function are presented in Table 3.

Next, we tested the activation function arctan. This time the number of hidden layers was 1–7, and the number of neurons was varied according to Table 4 In this case, the minimum cost found on the test batch was 0.31 at an accuracy of 95.67% with seven hidden layers and 16 neurons on each layer. The results obtained for the arctan activation function are presented in Table 4.

The best result was obtained using an architecture with six hidden states, eight neurons on each hidden layer, and each layer (excluding the output layer, which had softmax) having the arctan activation function. The accuracy of classes (in this case, on the test group) was 98.30%.

In the second stage, optimization, validation, and increase of generalization capacity, the database used in stage one was divided into 80% for training and 20% for testing (another test was done for ordering the datasets into 80% for training, 10% for validation, and 10% for testing) using the SGD optimization algorithm.

The algorithms presented in this article were developed in the LabVIEW 2020 SP1 programming environment with a 17-inch high-performance portable computing system with an Intel processor, four cores @ 3.2 GHz, 16 GB DDR3 RAM @ 1600 MHz, 256 GB SSD, 1 TB HDD, and high-performance dedicated video card, optimized for computer graphics, with K-complex detection being offline for optimized for computer graphics to train and test the MLP-CNN neural network with Wigner–Ville. In the future, we hope to develop a portable system for online analysis based on an FPGA architecture with an Intel Core i7 processor (3.1 GHz) or Coral Dev Board that can be used for a machine learning embedded system; again, the software will be developed in the Pytorch framework.

The final part of a deep network is represented by one or more fully connected multilayer perceptron (MLP) layers that perform the classification part, using as inputs the outputs of the initial convolutional part (characteristics automatically extracted by it) obtained for each applied signal network input.

The optimizer is the algorithm that decides in which direction and how strongly to change the weights in the network. In order to adjust the parameters of a network (weights and displacements), an optimizer must be used that decides the modification strategy according to the gradients obtained with the help of the loss function. The algorithm used is Calibrated Stochastic Gradient Descent (CSGD). The optimizer requires specifying the size of the optimization step, also called the learning rate, which in our tests will be kept constant at 10^−5^.

It is, practically speaking, an iterative optimization process that aims to reduce the error (respectively the difference between the expected output and the one obtained in that iteration), estimated using a cost (or loss) function. In this way, those inputs and characteristics that are the most relevant for obtaining the desired output are enhanced.

This experiment resulted in a classification accuracy of 98.3%, recall of 0.96, and microaverage F1 score of 0.97. The confusion matrix is shown in Table 5 and the classification report is given in Table 6.

This was to be expected as the cost does not provide information on accuracy. The latter is calculated according to the formula:

(14) $$accuracy=\frac{number\;of\;cases\;classified\;correctly}{number\;of\;total\;cases}$$

and the cost function used is binary cross entropy and behaves according to the following law:

(15) $$ℒ=-\frac{1}{n}\sum_{i=1}^{N}y_{i}log\left(\hat{y}\right)+\left(1-y_{i}\right)log\left(1-\hat{y}\right).$$

**Figure 12:**
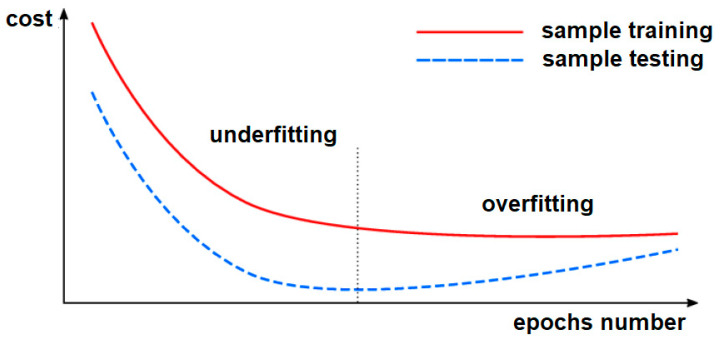
Illustration of underfitting and overfitting.

In the field of neural networks, there are two phenomena that can occur during training: underfitting and overfitting. The first refers to when learning is stopped too quickly compared to the number of epochs. As can be seen from Figure 12, underfitting occurs when the cost is still decreasing, both for the training group and for the test group, but a minimum cost has not been reached. Therefore, the subtraining area is characterized by low accuracy in the learning and testing groups. Overfitting is the extreme opposite of underfitting. This time the network is left to learn for a large number of eras. In this case, parameters were found that fit the drive data almost perfectly. The problem is that the network did not learn a real connection between the signals, but only managed to map the input data to the corresponding output values. In this case, the cost per test batch increases because the network is not robust and does not manage new data well.

Analyzing the results presented in this article, we can say that in no variant of the tested architecture was there overfitting. However, the rule of saving the model was: keep the one that has the lowest cost on the test lot. Thus, all results presented are not affected by either underfitting or overfitting.

The computational time required to extract the characteristics of spectrograms using MLP-CNN was 1.26 s. The total training time required for the EEG spectrograms was 13 min. The trained model classified K-complexes’ shape in 5 s for offline analysis and classifications.

To compare the results obtained with the proposed method, we referred to the following articles [69,70,71,72,73], which have as their theme the automatic detection of the K-complex. The comparative results are presented in Table 7.

## 4. Discussion and Conclusions

The present study was spurred by the need to develop a definition of a methodology for the synthesis of imposed structure detectors with applications in the detection of K-complexes using time–frequency representation. Furthermore, before drawing any conclusions, we must recall the main outcomes, from which we can highlight some future research directions. Initially, we presented the notion of time–frequency representation, insisting on the distribution of energy in the Cohen class. We have presented some practical problems related to their use, such as the presence of interference terms that are harmful to the interpretation of results.

Among other difficulties that may arise, we have been particularly interested in the large amount of computing that occurs in the analysis of long signals. Thus, after emphasizing the potential of recursive algorithms for the rapid calculation of time–frequency representations, we presented in a homogeneous and unified manner the direct and indirect recursive properties of the Cohen class.

Furthermore, we expanded their distribution by the reallocation method. Then, we made the first strategic choice in defining a methodology for the design of imposed structure detectors using a deep neural network. The aim of the present study was to select the best detection test for a given pattern, as far as the definition of an optimal criterion is concerned. To determine the decision rule, our choice was a deep neural network with arctan-type activation function, due to its consistency, which is a guarantee of performance. The method allowed us to present an answer to this problem, leading to the analytical determination of the best criterion, for which the detector for the obtained K-complexes has a minimum probability of error. Finally, the time–frequency plan, which is an optimal space for analyzing the configuration of some detectors, made it possible to verify the method. An original presentation of the potential of the Cohen class in solving certain detection problems with an imposed structure is provided, which gives the representation a decisive role in the decision-making process. In the final stage, different deep neural network architectures were tested. During the tests, the hyperparameters of the system were modified. More precisely, we experimented with various values for the number of neurons and layers, and various activation functions—tanh, arctan, and sigmoid. These three activation functions were chosen because they have an S shape, which increases the separability of the input data. The best result was obtained using an architecture with six hidden states, eight neurons on each hidden layer, and with each layer (excluding the output layer, which had softmax) having the arctan activation function. The accuracy of classes (in this case, in the test group) was 98.30%.

The limitations of this study were: (a limitation of all sleep EEG studies) recorded EEG signals represent the sum of the electrical activity in large areas of the brain, which can lead to an inaccurate location; to improve the performance of the proposed method it is useful to identify K-complexes from multi-electrode data; EEG recordings for somnology are often not of a quality requiring the use of filters, which can introduce the smoothing of the EEG signal, which is interpreted as uniformity of brain activity during sleep; in most cases, public databases for brain activity during sleep are not annotated, which makes it difficult to train the neural network; future studies with larger annotated sleep EEG databases will be needed to assess and compare the robustness of our method at each stage of sleep, as access to specialized somnology clinic databases is currently very limited due to GDPR legislation; one direction of increasing the performance of our method is the fine adjustment of the analysis and recognition parameters from one subject to another, taking into account the individual differences of the subjects and the properties of the K-complex.

Given both the theoretical and practical knowledge gained from the research presented in this article, and the software developments made to validate the theoretical results, we believe that they can be successfully addressed in the future: analysis and implementation of theoretical research that can lead to a link between time–frequency analysis and the SRM (Structural Risk Minimization) principle; optimizing the reconstruction property of Wigner–Ville representations, starting from a partial, but not random, knowledge of it, to remove the signals from the noise and the exact representation of the K-complex and the sleep spindle, synthesizing a linear detector that operates only on independent linear components and can fully process Wigner–Ville representations and the realization of a portable hardware (e.g., a Coral Dev Board can be used for a machine learning embedded system) and software system for the analysis, recognition, and classification of a K-complex and sleep spindle in real time so that this system can be used in patients’ homes (home care).

Finally, the methodology presented in this paper has been validated for an application with a certain degree of difficulty, namely, the detection of the K-complex in the sleep electroencephalogram. The proposed solution is among the performance solutions described in the literature. In addition, these results may be improved by correlating information from multiple electroencephalogram records.

## Figures and Tables

**Table 1:** Cohen class frequency–time representations and associated core functions.

Φ(ξ,τ)	$C_{x}\left(t,\nu ,\Phi \right)$	Distribution
1	$\int_{-\infty }^{\infty }f\left(t+\frac{\tau }{2}\right)\cdot f^{*}\left(t-\frac{\tau }{2}\right)\cdot e^{-i\cdot 2\pi \cdot \nu \cdot \tau }d\tau$	Wigner-Ville
$\frac{\sin\left(\pi \xi \tau \right)}{\pi \xi \tau }$	$\int_{-\infty }^{\infty }\left[\frac{1}{\left|r\right|}\int_{t-\frac{\tau }{2}}^{t+\frac{\tau }{2}}f\left(s+\frac{\tau }{2}\right)\cdot f^{*}\left(s-\frac{\tau }{2}\right)ds\right]\cdot e^{-i\cdot 2\pi \cdot \nu \cdot \tau }d\tau$	Born-Jordan
$e^{\frac{-{\left(\pi \xi \frac{\tau }{\sigma }\right)}^{2}}{2}}$	$\int_{-\infty }^{\infty }\int_{-\infty }^{\infty }\frac{\sigma }{\left|r\right|}\cdot e^{-2\sigma^{2}{\left(s-t\right)}^{2}\cdot \tau^{2}}\cdot f\left(s+\frac{\tau }{2}\right)\cdot f^{*}\left(s-\frac{\tau }{2}\right)e^{-i\cdot 2\pi \cdot \nu \cdot \tau }dsd\tau$	Choi-Williams
$A_{h}^{*}\left(\xi ,\tau \right)$	${\left|\int_{-\infty }^{\infty }f\left(s\right)\cdot h^{*}\left(s-t\right)\cdot e^{-i\cdot 2\pi \cdot \nu \cdot s}ds\right|}^{2}$	Spectrograma

**Table 2:** The results obtained for the tanh activation function.

Number of Neurons	Training		Testing	
	Cost	Accuracy	Cost	Accuracy
8	0.57	75.57	0.35	87.05
16	0.58	72.00	0.32	90.18
32	0.58	73.29	0.46	88.62
64	0.56	74.56	0.34	87.18
128	0.54	74.23	0.38	85.65
256	0.55	75.83	0.38	89.97

**Table 3:** The results obtained for the sigmoid activation function.

Number of Neurons	Training		Testing	
	Cost	Accuracy	Cost	Accuracy
8	0.52	72.87	0.35	94.65
16	0.52	74.00	0.38	84.65
32	0.58	72.89	0.41	84.62
64	0.53	74.00	0.38	93.23
128	0.58	64.25	0.38	82.35
256	1.55	52.63	0.75	90.17

**Table 4:** The results obtained for the arctan activation function.

**Number of Neurons**	**Training**		**Testing**	
	**Cost**	**Accuracy**	**Cost**	**Accuracy**
8	0.67	67.87	0.35	98.30
16	0.67	64.53	0.31	95.67
32	0.55	72.79	0.31	92.62
64	0.58	74.31	0.33	88.53
128	0.52	78.25	0.34	87.38
256	0.55	71.13	0.35	94.17

**Table 5:** The confusion matrix from MLP-CNN with Wigner–Ville spectrogram classes.

		Predicted Label		
		Background Noise	Single EEG Signal	Two EEG Signals
True Label	Background Noise	1.00	0.00	0.00
	Single EEG Signal	0.00	0.96	0.06
	Two EEG Signals	0.00	0.93	0.57

**Table 6:** Classification report for MLP-CNN with Wigner–Ville spectrograms.

Classes	Precision	Recall	F1 Score
Background Noise	1	1	1
Single EEG Signal	0.99	0.98	0.98
Two EEG Signals	0.98	0.97	0.97
Average/total	0.98	0.95	0.96

**Table 7:** Comparison of methods used in the automatic detection of K-complexes.

Reference	Year	Method Used	Detection Result
[69]	2015	Wavelet	81.57%
[70]	2019	Fractal Time–frequency	97.00%
[71]	2021	SVM	97.70%
[72]	2018	Deep Learning	94.00%
[73]	2020	Multitaper Method	93.70%
Proposed Method	2021	Time–Frequency and Deep Neural	98.30%

## Data Availability

Anonymized records obtained from a somnology clinic were used, and the resulting software application is to be tested in the same clinic.
